# Land-use and agriculture in Denmark around year 1900 and the quest for EU Water Framework Directive reference conditions in coastal waters

**DOI:** 10.1007/s13280-021-01536-8

**Published:** 2021-03-18

**Authors:** Bent T. Christensen, Birger F. Pedersen, Jørgen E. Olesen, Jørgen Eriksen

**Affiliations:** Department of Agroecology, Aarhus University, AU-Foulum, Blichers Allé 20, P.O. Box 50, 8830 Tjele, Denmark

**Keywords:** Abuse of N balances, Agricultural management, Coastal water eutrophication, EU water framework directive, Historic parish-level land-use

## Abstract

The EU Water Framework Directive (WFD) aims to protect the ecological status of coastal waters. To establish acceptable boundaries between good and moderate ecological status, the WFD calls for reference conditions practically undisturbed by human impact. For Denmark, the nitrogen (N) concentrations present around year 1900 have been suggested to represent reference conditions. As the N load of coastal waters relates closely to runoff from land, any reduction in load links to agricultural activity. We challenge the current use of historical N balances to establish WFD reference conditions and initiate an alternative approach based on parish-level land-use statistics collected 1896/1900 and N concentrations in root zone percolates from experiments with year 1900-relevant management. This approach may be more widely applicable for landscapes with detailed historic information on agricultural activity. Using this approach, we find an average N concentration in root zone percolates that is close to that of current agriculture. Thus, considering Danish coastal waters to be practically unaffected by human activity around year 1900 remains futile as 75% of the land area was subject to agricultural activity with a substantial potential for N loss to the environment. It appears unlikely that the ecological state of coastal waters around year 1900 may serve as WFD reference condition.

## Introduction

The EU Water Framework Directive (WFD 2000) represents a crucial step in regulating the ecological quality of coastal waters (the water body stretching 1.85 km from the coastline). It aims to protect the biological quality and to reestablish good ecological status for surface waters adversely affected by human activity. Since no universal quality standard applies to all coastal waters, the WFD calls for a defined status of a given water body that corresponds to no or only very minor anthropogenic impacts compared to that of undisturbed conditions, termed reference conditions. The reference condition is a key element in establishing the important boundary between good and moderate status of a given coastal area and in any regulation of anthropogenic inputs (e.g. Skarbøvik et al. 2020). The guidance document associated with the WDF (WDF-CIS-5 2003) outlines a hierarchical approach for deriving reference conditions in coastal waters: existing undisturbed sites, historical information, models, and expert judgements. However, attempts to define reference conditions most often combine these approaches.

The ecological status around year 1900 has repeatedly been promoted as defining reference conditions in Danish coastal waters (Baattrup-Pedersen et al. 2008; Andersen et al. 2011; Kaas et al. 2015; Jensen 2017). It is assumed that the anthropogenic impact at that time was negligible as judged from historic distributions of eelgrass (*Zostera marina* L.) and concentrations of chlorophyll a derived from water transparency measured by Secchi depth. However, the variability of eelgrass abundance within a given coastal area (e.g. Krause-Jensen et al. 2005; Kuusemäe et al. 2016) may compromise eelgrass as universal ecological indicator (Riemann et al. 2016). The observed links between Secchi depth, chlorophyll a, and nitrogen (N) concentrations prompted N levels in coastal waters as an indicator for ecological status (Nielsen et al. 2002; Henriksen 2009; Schernewski et al. 2015) even though no universal relationship exists between chlorophyll a and N concentrations (Carstensen et al. 2011).

Defining targets for N use in agriculture is of relevance to all EU member states and has attracted much attention in the Baltic area (e.g. Gustafsson et al. 2012; Wulff et al. 2014; Andersen et al. 2017; Skarbøvik et al. 2020). Indeed, it has very high priority in Denmark as 62% of the land area is currently subject to agricultural activity and the N load to coastal waters relates closely to runoff from land. Small watercourses, distributed across the land and draining to coastal areas with different ecological susceptibility, may lead to widely different regional reduction targets in N use. However, measurements of N concentrations in Danish streams around year 1900 are extremely few (Westermann 1898) and their representativeness uncertain. The N load of coastal waters in year 1900 therefore remains currently unknown.

Several European studies combine expert judgements and historical data with hindcast modelling to establish WFD reference conditions 100 to 150 years ago (Gustafsson et al. 2012; Hirt et al. 2014; Schernewski et al. 2015; Andersen et al. 2017), assuming insignificant anthropogenic N loads or involving historical N balances with the N surplus linked to N leaching from land. This may lead to substantial negative N balances (Hirt et al. 2014) with subsequent upscaling of negative to positive surplus to comply with model requirements (Gadegast et al. 2012). Negative N surplus certainly questions the feasibility of historic N balances in estimating N loss potential from agricultural land.

The nutrient status of Danish coastal water around year 1900 relies on current N contents in streams with negligible human impact and national N surplus for agriculture year 1900 (Kaas et al. 2015; Jensen 2017). Based on pristine catchments and estimates of national N surplus, this approach is open for criticisms as agricultural activity around 1900 affected 75% of the land area (Danmarks Statistik 1968).

Here we apply a critical perspective on the use of N balances to predict historic N loading of coastal waters for establishing N reduction needs to comply with WFD objectives. Further, we initiate an alternative approach that focuses on the source strength (i.e. N concentration in water leaving the root zone) using parish-level statistics on land-use (collected 1896/1900) and nitrate–N in percolates from field experiments with year 1900-relevant management. This allows for future calculations of N in water reaching Danish coasts in 1900 and for testing the relevance of using year 1900 as hallmark for WFD reference conditions.

## Historical aspects of Danish agriculture

The statistics on land-use and agriculture in this section relate to land under Danish administration before 1920. During 1861–1896, the area in agricultural use increased dramatically and accounted for 75% in 1896. The area in rotational cropping increased by 546 000 ha (Jensen 1988), and heathlands and sand dunes declined by 656 000 ha during 1850–1907 (Mortensen 1969). Bare fallow with vegetation-free soil subject to frequent tillage throughout a year was widespread (Christensen 1898). First introduced in Denmark in the 1850s, tile-drainage affected 26% of the agricultural area by year 1907 with 300 000 ha being drained during 1871–1881 (Jensen 1988).

### Crop production and N use

Crop production around 1900 differed markedly from that of current agriculture: crops with low yield potentials, inferior horse-drawn implements, high weed pressure, lack of chemical plant protection, and inefficient plant nutrient supply. Mineral N fertilizer use averaged 1 kg N ha^−1^ (Danmarks Statistik 1968), the main sources of N being animal manure and N_2_-fixing crops. The annual average (1900–1904) application of N in manure was 21 kg N ha^−1^ when corrected for 15% loss of N during feeding and 25% loss of N during manure storage (Danmarks Statistik 1968) but not accounting for loss of N after field application.

In 1896, storage tanks for liquid manure accounted to 28 000 (Iversen 1944) while manure heaps with roof covers were 16 500 in 1907. There was 237 000 farms and smallholdings with farmland and another 35 000 holdings without land (Christensen 1985). Thus, only a small proportion of the holdings had proper manure storages, facilitating substantial volatilization and leaching losses of manure-N. Field application of manure occurred typically during late summer, autumn and early winter, due to cultivation of autumn-sown crops, lack of manure storage capacity, and the availability of farm labour. Manures applied during this period provide a poor N-use-efficiency and substantial leaching potentials of mineral N present in the manure or mineralized from organically bound N outside the active growing season.

The grain yields for oats, barley, rye and wheat around 1900 averaged 1.4, 1.8, 2.0 and 2.8 t ha^−1^, respectively (Danmarks Statistik 1968). These yields align with those achieved during 1894–1904 in the Askov long-term field experiments, in plots kept unmanured for more than 120 years (Christensen et al. 2019), and in unmanured plots in ongoing organic farming experiments (Olesen et al. 2002; Shah et al. 2017). Hay production on rotational and permanent grassland (incl. meadows) yielded 2.4 and 2.7 t ha^−1^, respectively (Danmarks Statistik 1968). Even though contemporary textbooks prescribed generous use of liquid manure to meadows, permanent grasslands and grass-clover crops in rotation in late autumn and again in the spring (Christensen 1898), yield levels were below those obtained currently for rotational grass-clover grown under unmanured conditions (Christensen et al. 2019).

The typical crop rotation in 1900 was spring cereals (mainly oats) undersown with grass-clover, 3 to 5 years in grass-clover followed by 1 year in bare fallow, and finally autumn-sown cereals (mainly winter rye), and/or a root crop (Christensen 1898). Fertile soils supported more crops of spring-sown oats before soil nutrients were exhausted and a new grass-clover crop was established.

### Animal production

Animal husbandry also differed from current Danish agriculture for most production factors: livestock composition, feed quality and rate of feeding, grazing intensity and periods, and productivity per animal unit. Converted into livestock units (1 cow = 1 livestock unit; LU), the agricultural sector included 2.6 million LU in 1898 (Danmarks Statistik 1969) with 54% cattle, 16% pigs, 15% horses, 8% poultry, and 7% sheep and goats. Grass ingested in fresh condition dominated ruminant forage (49%), while root crops, hay and cereal straw accounted for 19%, 18%, and 13%, respectively (Danmarks Statistik 1968). Grazing accounted for more than 70% of the feed intake (Kristensen et al. 2015).

### Farm structure

The farm structure differed fundamentally from that of current Danish agriculture with most of the agricultural production currently concentrated on 10 000 farms. At that time, most farms were small in terms of acreage and production volume. Farm sizes were measured in hartkorn (Hkt), a unit that combines land area, land-use and soil quality providing an estimate of the production potential of individual farms. Smallholdings (< 1 Hkt) and smaller farms (1 to 8 Hkt) accounted in 1895 for 74% of the total agricultural production (Christensen 1985). Cattle herds encompassing 1 to 14 cows accounted for 70% of all cows and the average animal density in 1898 was 0.89 LU ha^−1^ on land under agricultural use (Danmarks Statistik 1969).

## The use and abuse of nitrogen balances

The use of historical N balances to establish WFD reference conditions implies an intimate link between N application and N leaching. Howarth et al. (1996) found a linear relationship between net anthropogenic N inputs (NANI; atmospheric deposition + fertilizer N application + agricultural N_2_ fixation + N in net food and feed imports) and N loss to coastal waters, the regional N fluxes in rivers averaging 25% of the calculated NANI. In that study, mineral N fertilizer dominated NANI. Hong et al. (2012) and Wulff et al. (2014) adopted the NANI concept to predict current riverine N export and estimate reduction targets at national scales. They concluded that NANI related closely to riverine nutrient fluxes and that meeting water quality targets may require substantial changes in the agricultural sector. However, Howarth et al. (2012) stated that the NANI approach, originally developed for very large regions, was likely to break down when applied to smaller watersheds. The NANI concept neglects changes in N residing in soil organic matter pools and that farm structure and field management affect crop N-use-efficiency. The latter is of particular importance when comparing agriculture with substantial differences in technology level.

**Fig. 1:**
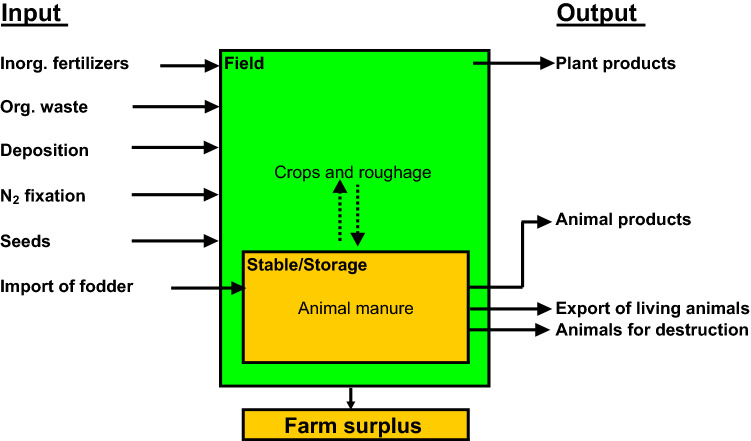
Farm-gate N balance approach

**Fig. 2:**
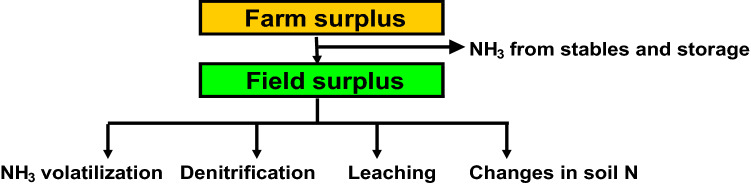
Distribution of farm-gate N surplus on ammonia volatilization from stables and manure storage, and the distribution of the resulting field N surplus

Calculations of N surplus used in hindcast models considers agricultural activity within a given catchment as one big farm (Fig. 1), thereby neglecting important internal N flows within the catchment and the volatilization of ammonia from animal housings and manure storage. Further, it ignores the use-efficiency of N inputs from animal manures and N_2_ fixation. A farm-gate balance does not account for the impact of management on the distribution of the field surplus N between leaching, ammonia volatilization, denitrification, and changes in the soil N pool (Fig. 2). However, setting up a historical field balance requires detailed information on inputs and outputs of N, and on N turnover rates at field level, information that is rarely available.

For current agriculture, the field N surplus and associated N leaching losses from the root zone shows little or no correlation (Blicher-Mathiesen et al. 2014, 2019; Eriksen et al. 2015; Hansen et al. 2015). Analysing 39 streams from catchments with between 0 and 90% of the area in agricultural use, Kronvang et al. (2015) found a good correlation between percentage of land in agriculture and the flow-weighted N concentration in streams without including the field N surplus. Chambers et al. (2012) reported similar results in a Canadian study. For Danish shallow lakes, Nielsen et al. (2012) showed a good relation between N concentration and percentage of land in agriculture while field N surplus showed no correlation. The lack of correlation between field N surplus and N leaching may reflect changes in the soil N pool and the dominating importance of field management.

Around year 1900, the N leaching most likely constituted a high proportion of the N surplus. Large areas of land had been included in rotational cropping in the preceding decades and there was a widespread use of bare fallow. Moreover, much of the arable land had recently been tile-drained and autumn application of manure enhanced N leaching losses. Drainage of waterlogged soil increases aeration and thereby the turnover of soil organic matter, promoting mineralization of N from the soil pool. Conversion of permanently vegetated land into arable rotation initiates a decrease in soil organic matter content that lingers on for decades. Whitmore et al. (1992) found that ploughing of permanent grassland reduced the soil N pool by up to 40% over a period of 20 years. Howden et al. (2010) found similar effects of cultivation. There is little doubt that in year 1900 significant losses in soil organic N occurred on areas that had been brought into cultivation and drained in the previous decades. Although the growing crops recovered part of the mineralized soil N, nitrate leaching most likely accounted for a substantial part of the decline as N mineralisation continued outside the growing season. There was a widespread use of spring-sown crops with manure applications in the autumn and leaving the ploughed soil bare during the autumn and winter periods. Neglecting the cultivation-induced loss of soil N may lead to negative N surplus as observed in previous studies (Gadegast et al. 2012; Hirt et al. 2014).

## Parish-level statistics (1896/1900)

Andersson and Arheimer (2003) applied a parish-level approach to establish historical N discharge from a catchment in South Central Sweden, including three land-use categories, historical information on land management, and model simulations of N leaching. Here we establish year 1900 estimates of nitrate–N in root zone percolates for eight land-use categories derived from historic parish-level statistics and N concentrations in percolates measured in well-defined field experiments with year 1900-relevant management.

From 1864 to 1920, the southern part of Jutland (Northern part of Schleswig) was under German administration. However, we obtained parish statistics for areas under Danish and German administration in 1896 and 1900, respectively, and thereby covers the current Danish territory. The parish boundaries have remained almost unchanged since year 1900 and thus the parish represents probably the most conservative land area unit. Each parish collected detailed information on land-use every 5 to 10 years. The present study relies on 1766 parish units.

**Fig. 3:**
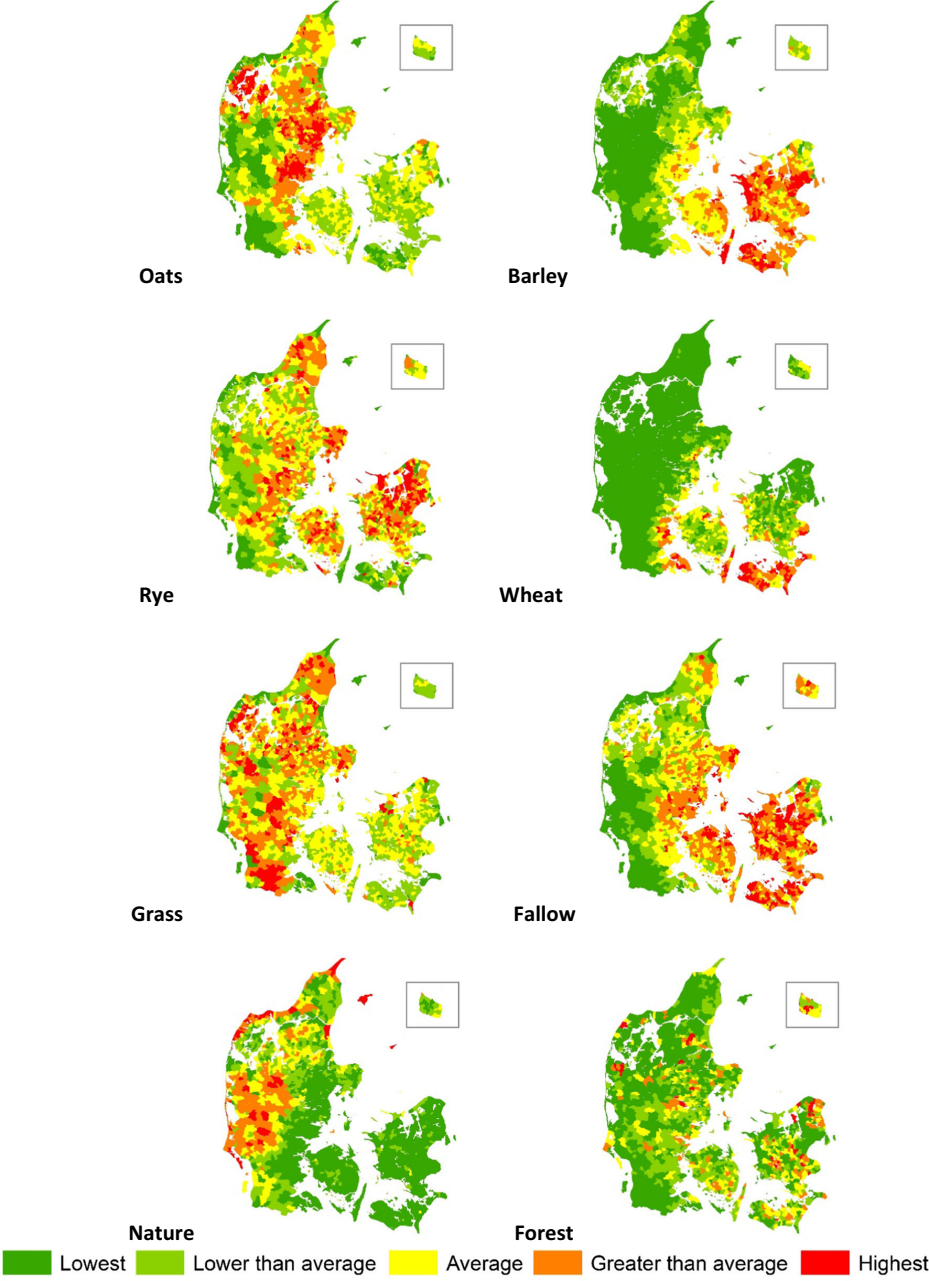
Relative abundance of oats, barley, cereal rye, wheat, grassland, fallow, nature (uncultivated land) and forest at parish level in 1896/1900

The Danish administration allocated the total area of each parish into 34 land-use categories (here coded DA, Table 1), while the German administration applied 51 categories (here coded TY, Table 2). We merged the two sets of data into 26 categories using the Danish categories as template (coded S, Table 3). Figure 3 shows the relative distribution of selected land-uses at the parish level. Next, we condensed the 26 S-coded land-use categories into eight DK-categories (Table 4) to align major land-uses with current year 1900-relevant N concentrations in root zone percolates.

**Table 1:** Land-use data collected in 1896 in parishes under Danish administration (3 880 000 ha; Statens Statistiske Bureau & Danmarks Statistik 1898)

Code	Land-use category	% of total area
DA1	Wheat	0.9
DA2	Cereal rye	7.6
DA3	Barley	7.4
DA4	Oats	11.6
DA5	Mixed cereals (mature)	3.1
DA6	Buckwheat	0.3
DA7	Pulses	0.2
DA8	Spurrey (mature)	0.2
DA9	Caraway and oil-seed rape	< 0.0
DA10	Seed production (clover, grass, beets, lupines)	0.1
DA11	Potatoes	1.4
DA12	Sugar beets and chicory	0.3
DA13	Carrots	0.2
DA14	Fodder beets	1.8
DA15	Green forage (mixed cereals, spurrey, lucerne)	1.3
DA16	Flax, hemp and tobacco	< 0.0
DA17	Garden crops	< 0.0
DA18	Black fallow (vegetation-free)	5.1
DA19	Black fallow (green manure before ploughing)	0.1
DA20	Semi-black fallow (early summer-crop)	1.4
DA21	Cultivated grass for hay	6.9
DA22	Cultivated grass for grazing	17.9
DA23	Meadows	6.0
DA24	Fens and commons	2.5
DA25	Moors and peatland	2.0
DA26	Hedgerows and shelters	0.2
DA27	Gardens and plant nurseries	0.9
DA28	Forest area (planted)	6.3
DA29	Forest area (unplanted)	0.7
DA30	Heathland	9.2
DA31	Sand dunes and shifting sands	1.1
DA32	Swamp, foreshores, stone fields etc.	0.4
DA33	Roads, building sites and storage areas	2.3
DA34	Lakes, ponds, streams (outside sea territory)	0.3
Total		100

**Table 2:** Land-use data collected in 1900 in parishes under German administration (390 000 ha; Engelbrecht 1907)

Category code	Land-use category	% of total area
TY1	Winter wheat	1.6
TY2	Spring wheat	< 0.0
TY3	Winter rye	5.6
TY4	Spring rye	< 0.0
TY5	Winter barley	< 0.0
TY6	Spring barley	4.4
TY7	Oats	8.9
TY8	Mixed cereals (winter)	< 0.0
TY9	Mixed cereals (summer)	2.1
TY10	Buckwheat	0.7
TY11	Peas	0.1
TY12	Fava bean	< 0.0
TY13	Vetch	< 0.0
TY14	Mixed cereals	0.4
TY15	Mixed pulses	< 0.0
TY16	Other types	< 0.0
TY17	Potatoes	1.0
TY18	Sugar beets	< 0.0
TY19	Fodder beets	0.6
TY20	Carrots	0.1
TY21	Fodder radish	0.1
TY22	Swedes	1.3
TY23	Field herbs and caddish	< 0.0
TY24	Other types	< 0.0
TY25	Winter rape and radish	< 0.0
TY26	Leindotter (Camelina sativa)	< 0.0
TY27	Flax	< 0.0
TY28	Other types	< 0.0
TY29	Clover (for forage)	1.0
TY30	Lucerne	< 0.0
TY31	Serradella	< 0.0
TY32	Spurrey	< 0.0
TY33	Seed production (clover, grass-clover)	4.2
TY34	Maize	< 0.0
TY35	Vetch	< 0.0
TY36	Lupines (for forage)	< 0.0
TY37	Mixed legumes	0.2
TY38	Mixed vegetables (for forage)	< 0.0
TY39	Mustard	< 0.0
TY40	Lupines	< 0.0
TY41	Mixed vegetables	< 0.0
TY42	Mustard	< 0.0
TY43	Fallow	3.3
TY44	Cultivated grass	25.0
TY45	Gardens and fruit plantations	0.7
TYG	Meadows	10.7
TYH	Pastures	11.1
TYI	Forests	3.6
TYJ	Buildings and yards	0.7
TYK	Uncultivated land	5.8
TYL	Roads and lakes, ponds, streams	6.7
Total		100

**Table 3:** DA and TY land-use categories and distribution merged into 26 S-categories

Code	Land-use category	Area (ha)	% of total area	DA code	TY code
S01	Wheat	41 428	1.0	1	1, 5
S02	Cereal rye	315 998	7.3	2	3
S03	Barley	301 057	7.0	3	6
S04	Oats	479 084	11.1	4	7
S05	Mixed cereals	129 831	3.0	5	2, 4, 8, 9
S06	Buckwheat	14 530	0.3	6	10
S07	Pulses	11 600	0.3	7	11, 12, 13, 14, 15, 16
S08	Spurrey	7641	0.2	8	32
S09	Caraway and rape	525	< 0.0	9	25, 39, 42
S10	Seed production	25 028	0.6	10	29, 30, 31, 33
S11	Potatoes	56 743	1.3	11	17
S12	Sugar beets	13 583	0.3	12	18
S13	Carrots	6 535	0.2	13	20
S14	Fodder beets	76 910	1.8	14	19, 21,22
S15	Green forage	51 277	1.2	15	23,24, 35, 36,37, 38, 40
S16	Flax, hemp and tobacco	293	< 0.0	16	27, 28
S17	Garden crops	37 549	0.9	17, 27	45
S18	Fallow	267 843	6.2	18, 19, 20	43
S19	Cultivated grass	1 053 714	24.5	21, 22	44
S20	Meadows, fens and commons	407 621	9.5	23, 24	G, H
S21	Moors, peat- and heathland	587 555	13.7	25, 30, 31, 32	K
S22	Forest	295 580	6.9	26, 28, 29	I
S23	Roads and building sites	88 336	2.1	33	No code
S24	Lakes, ponds and streams	13 086	0.3	34	No code
S25	Buildings and yards	2967	0.1	No code	J
S26	Roads and water areas	17 441	0.4	No code	L
Total		4 303 762	100

**Table 4:** DK-model codes merged from S codes: Land-use, areas and nitrate–N concentrations in roots zone percolates for each DK-model category. Area adjusted to the total area of Parish maps 1900

DK code	Land-use and area				Nitrate–N concentration
	Land-use category	1000 ha	%	S land-use codes	mg N L^−1^
DK-1	Winter crops	357	8.3	1,2	18
DK-2	Spring crops	982	22.8	3,4,5,6,7,8,9,16,17	13
DK-3	Grass	1538	35.7	10,15,19,20	9
DK-4	Roots	154	3.6	11,12,13,14	12
DK-5	Fallow	268	6.2	18	20
DK-6	Nature	588	13.7	21	1
DK-7	Forest	296	6.9	22	2
DK-8	Other land-use	122	2.8	23,24,25,26	0
Total		4305	100		

## Estimates of N concentrations in root zone percolates

Results from field experiments with year 1900-relevant management allowed us to link specific and well-known management with N concentrations in root zone percolates from agricultural soils. For forest-covered areas, we relied on N concentration in soil solutions retrieved from the bottom of the root zone.

The N_2_ fixation depends on crop species and competition with companion non-fixing plants. Average annual yields of grass-clover leys grown during 1907–1922 in animal-manured plots of the Askov long-term field experiments was 4.8 t DM ha^−1^ (Iversen and Dorph-Petersen 1951) of which 42% was clover biomass. Using an empirical model (Høgh-Jensen et al. 2004) for grass-clover leys, N_2_ fixation ranged 117 to 183 kg N ha^−1^. However, the fate of this N remains difficult to quantify.

Mineralization of organically bound N outside the growing season represents a substantial potential for N leaching. Following unfertilized grassland, terminated in the spring and then seeded to spring barley, Eriksen et al. (2008) found an annual flow-weighted nitrate concentration of 36 mg N L^−1^ in drainage following the barley crop. Leaching of N can be substantial from grasslands subject to long grazing periods. Around year 1900, grasslands received liquid manure in the early spring and after having delivered a first cut of hay, grazing continued until late autumn (Christensen 1898) with urination by grazing animals creating locally very high inputs of mobile N. For a 4-year old grass-clover field, subject to grazing from late April to late October, Hansen et al. (2012) estimated that urination affected one-third of the area, giving rise to 23 mg N L^−1^ in the root zone percolate. When exposed to a grass cut in the spring and then subjected to grazing, the N concentration was 19 mg N L^−1^.

A study of organic crop rotations was initiated in 1997 at three Danish sites varying in climate and soil type (Olesen et al. 2000). The rotation was grass-clover ley, winter cereals, spring cereals, and either a potato or a grain legume crop. Animal manure was applied at an average annual rate of 70 kg N ha^−1^ (~ 0.7 LU ha^−1^) and nitrate leaching measured at the bottom of the root zone (Askegaard et al. 2011). The flow-weighted nitrate concentration in percolates averaged 12 mg N L^−1^ and was remarkable constant across sites and three rotation cycles. However, amounts of N lost by leaching differed between sites as percolation ranged 238 to 637 mm. Eriksen et al. (1999) provide results from an organic rotation addressing cattle production. Nutrients added in liquid and bedding-rich farmyard manure and from grazing cattle corresponded to 0.9 LU ha^−1^. The rotation was spring barley undersown with grass-clover, two years with grass-clover, barley/pea, winter wheat and beetroots. The grass-clover was cut once early in the growth period and then exposed to grazing. Except for lack of bare fallow, the rotation and its management, including nutrient load, corresponded well with a year 1900 scenario. The average flow-weighed nitrate concentration in water leaving the root zone was 13 mg N L^−1^ for spring-sown crops (barley undersown with grass-clover and barley/pea mixture), 18 mg N L^−1^ for winter wheat, 9 mg N L^−1^ for grass-clover (first and second year grass-clover), and 12 mg N L^−1^ for the root crop. These values are associated with DK-codes 1 to 4 in Table 4.

Soils under bare fallow show significant N leaching losses. For unmanured bare fallow, Thomsen et al. (1993) found an annual average N leaching over a four-year period of 104 kg N/ha, nitrate in the leachate being 21 mg N L^−1^. Experiments with permanent fallow, established in 1870 at Rothamsted Experimental Station, showed a nitrate–N concentration of 19 mg N L^−1^ when averaged over seven years of fallow (Addiscott, 1988). For fallow (DK-5), the nitrate–N concentration was set to 20 mg N L^−1^ (Table 4).

We apply a concentration of 1 mg N L^−1^ for non-forest areas under natural vegetation (DK-6). We assume that in areas without net-gain in standing vegetation biomass and soil N storage, the leaching of N reflects deposition of N, and that N_2_ fixation and denitrification balance out. The first systematic measurements of N in rainwater, made in the period 1921–1926 at four research stations across the country, showed an annual deposition of N in ammonium and nitrate ranging 6 to 11 kg N ha^−1^ (Hansen 1931).

For N lost from forest (DK-7), we rely on studies of forests with different size and age (Callesen et al. 1999; Hansen et al. 2007). The average concentration of N in soil solutions extracted from 75 to 100 cm soil depth in forests with an area of < 10 ha, 10–50 ha and > 50 ha was 3.0, 2.0 and 1.3 mg N L^−1^, respectively. For a coniferous stand < 45 years grown on a nutrient poor location, the N concentration in 90 cm soil depth ranged 0.5 to 1 mg N L^−1^. Percolates from deciduous forests on a more nutrient rich locality varied around 4 mg N L^−1^, generally with the smallest values under younger stands. Concentrations of N increased with increasing stand age on more nutrient rich soils. The value for forest was set to 2 mg N L^−1^ (Table 4).

For other land-use (DK-8), we do not estimate a concentration as this land-use code categorizes as point sources or recipients.

## Reflections and reservations on root zone estimates

The N concentrations for the land-use categories autumn- and spring-sown crops, grass-clover and root crops are from well-monitored field experiments under organic farming with an animal stocking density of 0.9 LU ha^−1^. Although organic cropping excludes mineral fertilizers and chemical crop protection, current crop varieties with animal manure applied in the spring are superior compared to those used in year 1900 with most of the manure applied in periods with large potentials for N leaching. The increased N-use-efficiency associated with modern crops grown on well-drained soils and spring application of manure leads to higher yields than obtained year 1900. The higher N-use-efficiency in current organic field experiments may underestimate N leaching in year 1900 agriculture. However, an inferior quality of manure associated with year 1900 animal husbandry may balance this discrepancy. Moreover, cultivation of native land and tile draining during 1860 to 1900 introduced a large and long-lasting decrease in soil N storage, adding an annual background leaching loss of 10 to 100 kg N ha^−1^ to that associated with management practised year 1900.

Based on historic land-use and measurements in current year 1900-relevant crop rotations, the mean annual nitrate concentration in root zone percolates from Danish agriculture becomes 12 mg N L^−1^. This is comparable to concentrations of 14 mg N L^−1^ determined in the Danish Agricultural Catchment Monitoring Program (LOOP) during 2004–2016 (Blicher-Mathiesen et al. 2019) but lower than the 28 mg N L^−1^ found for 1990–1994. For the south and central Sweden, similar results were found for agricultural soils when comparing nitrate leaching in the late part of the 19th century to that in the 1980s (Hoffmann et al. 2000; Andersson and Arheimer 2003).

Our study excludes leaching losses of ammonium and organically bound N. The concentration of ammonium in drainage water collected during 1971–1991 from five loamy soils in agricultural use averaged 0.12 mg N L^−1^ (Simmelsgaard 1996). Studies on root zone percolates from two sandy soils under grass-clover crops showed average ammonium and organic N concentrations of 0.05 and 1.4 mg N L^−1^, respectively (Vinther et al. 2006). Thus, we consider that the concentrations of these forms of N remain inferior compared to those of nitrate–N.

## Perspectives

Using Denmark as a case study, we find that the use of parish-level land-use statistics and field experiments with year 1900-relevant management initiates a valid alternative approach to establish the export of N from land to coastal waters. Combined with hydrological models on catchment scales and using year 1900 precipitation patterns and retention of N in landscape elements, this may provide realistic estimates of the historic N load to individual coastal waters. We venture that this approach may be more widely applicable for catchments with detailed historic information on agricultural activity. However, in accordance with previous studies (Clarke et al. 2003; Henriksen 2009; Topcu et al. 2011; Duarte et al. 2015; Skarbøvik et al. 2020), we question the relevance of reference conditions based on historic catchment-scale N balances, crude categories of land-use, and hindcast modelling.

Denmark has since 1985 implemented a number of actions plans to reduce ammonia volatilization and nitrate leaching from agricultural activities (Dalgaard et al. 2014). Since 1990, this reduced the N input from diffuse land sources to coastal waters by 43% and resulted in significant declines in concentrations of N and chlorophyll a (Riemann et al. 2016). However, a non-linear response of N reduction to improvement in water quality may be expected (Duarte et al. 2009, 2015; Riemann et al. 2016) due to release of nutrients accumulated in landscape elements and sediments, change in catchment hydrology, and other environmental changes whether natural or induced by human activities.

The WFD (2000), adopted as national legislation in Denmark in 2003, requires reference conditions that represent no or very minor impacts from human activity to be established for coastal waters. We submit that considering year 1900 as a period where Danish coastal waters were almost unaffected by human activity remains unsupported as 75% of the land area was subject to agricultural activity with a substantial potential for N leaching losses to the aquatic environment. We conclude that the ecological state of coastal areas around year 1900 is unlikely to serve as WFD reference conditions.

Duarte et al. (2009, 2015) challenged central elements in the WFD, including the concept that a sufficient reduction of N use on agricultural land automatically return the ecological state of coastal waters to that existing in some idealized pre-perturbation period. Duarte et al. (2009) stated that this expectation is as likely as the existence of Neverland. We agree that return to Neverland cannot be an ambition but remains an illusion.
